# Exploring the Dynamic Invasion Pattern of the Black-Headed Fall Webworm in China: Susceptibility to Topography, Vegetation, and Human Activities

**DOI:** 10.3390/insects15050349

**Published:** 2024-05-13

**Authors:** Fan Shao, Jie Pan, Xinquan Ye, Gaosheng Liu

**Affiliations:** College of Forestry, Nanjing Forestry University, Nanjing 210037, China; shaofan187@163.com (F.S.); xinquan@njfu.edu.cn (X.Y.); laugousing@163.com (G.L.)

**Keywords:** fall webworm, invasion dynamic, susceptibility, standard deviation ellipse, spatial autocorrelation

## Abstract

**Simple Summary:**

In recent years, the damage inflicted upon vegetation by the fall webworm (FWW), *Hyphantria cunea* (Drury) (Lepidoptera: Erebidae: Arctiidae), has escalated globally. The black-headed fall webworm has extensively invaded regions in Europe and Asia. In China, scholars have conducted extensive research on the fall webworm. However, there has been limited research on the dynamics of invasion by the black-headed form of the fall webworm and the comprehensive interplay of various factors affecting its spread. Therefore, this study focuses on the dynamics of invasion by the black-headed form of the fall webworm and the combined influence of various factors affecting its spread. The plan is to map the invasion road map of Autumn Dictyophora melanocephala since its introduction into China and summarize the effects of terrain, vegetation, and human factors on the temporal and spatial changes of the species.

**Abstract:**

The fall webworm (FWW), *H. cunea* (Drury) (Lepidoptera: Erebidae: Arctiidae), is an extremely high-risk globally invasive pest. Understanding the invasion dynamics of invasive pests and identifying the critical factors that promote their spread is essential for devising practical and efficient strategies for their control and management. The invasion dynamics of the FWW and its influencing factors were analyzed using standard deviation ellipse and spatial autocorrelation methods. The analysis was based on statistical data on the occurrence of the FWW in China. The dissemination pattern of the FWW between 1979 and 2022 followed a sequence of “invasion-occurrence-transmission-outbreak”, spreading progressively from coastal to inland regions. Furthermore, areas with high nighttime light values, abundant ports, and non-forested areas with low vegetation cover at altitudes below 500 m were more likely to be inhabited by the black-headed FWW. The dynamic invasion pattern and the driving factors associated with the fall webworm (FWW) provide critical insights for future FWW management strategies. These strategies serve not only to regulate the dissemination of insects and diminish migratory tendencies but also to guarantee the implementation of efficient early detection systems and prompt response measures.

## 1. Introduction

The invasive species was defined by Reaser et al. [1] as, “with regard to a particular ecosystem, a non-native organism whose introduction causes, or is likely to cause, economic or environmental harm, or harm to human, animal, or plant health”. The invasion of new areas can disrupt the abundance and diversity of local species, increase the risk of native species extinction, and lead to a significant loss of human or financial resources in the invaded area [2,3]. In addition, as globalization and the expansion of global trade continue to grow, it is anticipated that the risk of invasive species infiltrating countries where they have not previously existed will also rise [4,5]. Consequently, it is imperative to restrict the entry or proliferation of invasive species into new regions. The most effective approach to limiting the spread of invasive species is through early detection followed by prompt and effective control or eradication measures [6]. Many countries have established registries of regulated species based on comprehensive risk assessments, which involve prohibiting the importation and trade of these organisms [7]. Nevertheless, the ingress of invasive organisms into new regions shows no signs of abating [8]. This phenomenon can be attributed to the introduction of invasive species into new areas driven by various factors. Different invasive species exhibit variations in their predominant influencing factors for propagation [6].

China’s vast territory and diverse habitats make it highly susceptible to invasive species. The rapid expansion of international trade has not only contributed to substantial economic growth but also triggered and accelerated biological invasions within China [9]. According to the Report on the State of the Ecology and Environment in China 2020, as far back as 2001, China had already recorded the introduction of 380 invasive alien species. By 2020, the number had dramatically surged, with more than 660 invasive alien species identified, resulting in an estimated annual economic loss exceeding $18.9 billion. In recent years, China has made significant progress in preventing and controlling alien invasive species by issuing a catalog of priority-managed alien invasive species and implementing localized control measures. Significant achievements have been attained in the control of invasive species such as *Cydia pomonella*, *Brontispa longissima*, *Eichhornia crassipes*, and *Mikania micrantha*, effectively mitigating their impacts on the ecological environment [10,11]. However, certain invasive species such as *H. cunea* and *Bursaphelenchus xylophilus* continue to proliferate within China, showing no discernible decline in their ecological impacts [12,13,14]. Therefore, to safeguard the ecological environment and mitigate ecological losses, it is imperative to intensify research efforts on invasive species.

The fall webworm (FWW), *Hyphantria cunea* (Drury) (Lepidoptera: Erebidae: Arctiidae), is one of the highly successful and rapidly spreading invasive species with global implications [15] and has been designated as China’s initial invasive insect species [16]. This insect has demonstrated strong adaptability to high temperatures, with the larva capable of withstanding temperatures up to 40–50 °C, as well as surviving at a minimum temperature of −23 °C during the pupal stage [17,18,19]. In the native United States, this pest is commonly found on wild trees but rarely poses a threat to economically important tree species [15]. Since its invasion in Europe and Asia, especially in China, it has demonstrated great adaptability and has become a major defoliator of fruit and street trees [20]. Recent studies also suggest great potential for extending the reach beyond temperature limits [21] and for evolutionary adaptation [22], making the elimination of the FWW challenging. Therefore, there is an urgent need to comprehensively explore the invasion dynamics of the FWW and the impacts of various factors influencing its spread to restrict its continued expansion into new areas.

Climate change is often considered a key factor influencing insect population dynamics [23]. Global warming is likely to exacerbate the ongoing and anticipated challenges posed by plant-eating pests, promote pest population growth, increase outbreak frequency, and facilitate the geographic expansion of many pest species [24]. Topographic factors play an important role in influencing insect population dynamics. On one hand, as altitude increases, the temperature decreases, leading to a reduction in insect populations [25]. On the other hand, high mountains serve as geographical barriers that can slow down or even prevent the spread of insects [26]. Vegetation unquestionably holds paramount significance for herbivorous insects, serving as their indispensable hosts essential for their survival [27]. Human activities, such as timber trading and commerce, can accelerate the spread of insects [28,29]. Clearly, these factors will more or less influence the spread of pests. To effectively prevent or manage these pests, studying the impact of these factors on them is crucial. Currently, research on the impact of climate change on the spread of the FWW has yielded encouraging results. Besides the current epidemic areas, potential suitable habitats for the FWW are identified in the eastern and southern regions of China, as well as in Shaanxi, Shanxi, Chongqing, Sichuan, Guangxi, and other areas [30]. However, there is currently a lack of comprehensive analysis regarding the combined impact of anthropogenic, topographic, and vegetative factors on the spread of the FWW. Therefore, this paper aims to explore the integrated impacts of anthropogenic, topographic, and vegetative factors on the dissemination of this pest within climates conducive to FWW survival.

The primary methods for studying the spatial distribution of species include spatial autocorrelation [31,32], and standard deviation ellipse [33]. The spatial autocorrelation, as measured by the Moran Index, is primarily used to evaluate the spatial differentiation of research subjects by identifying both global and local spatial correlation and clustering characteristics within the data [34,35]. It could effectively illustrate the clustering patterns of nighttime lights and fractional vegetation cover within the country. This clustering pattern, overlaid with the locations of fall webworm epidemic areas, can be used to explain and examine the impact of both human and natural factors on the spread of the fall webworm. The standard deviation ellipse (SDE) serves as an effective method to accurately reveal the spatial distribution attributes of geographical elements within spatial statistics. The eccentricity of the spatial distribution analysis provides insights into the research object’s pattern [36,37]. The application of this method enables a precise quantification of the spread trend of fall webworms. In summary, we employed the spatial autocorrelation method to analyze the impact of human and vegetation factors on the spread of the FWW. The SDE method, combined with the propagation path, was used to analyze the spread trend of the FWW in different periods in China.

In this study, we collected the black-headed FWW occurrence data from 1979 to 2022 to analyze the invasion dynamics of the black-headed FWW over the past four decades in China and determine the predominant factor influencing its spread under the conditions of a suitable climate for its survival. Our objectives were to explore the following questions: (1) What alterations have occurred in the transmission pathway of the black-headed fall webworm since its introduction to China, and what dynamic invasion patterns has it manifested? (2) In the future, under the combined influence of terrain, vegetation, and anthropogenic factors, how is the black-headed FWW more likely to occur in China? 

## 2. Materials and Methods

### 2.1. Study Area

China (Area: 9.60 × 10^6^ km^2^) is located in the east of Asia along the west coast of the Pacific Ocean (73°33′–135°05′ E, 3°51′–53°33′ N) [38]. The nation experiences obvious seasonal variations in both temperature and precipitation, characterized by four distinct seasons, featuring cold winters and relatively dry summers across most regions. With complex topography, diverse climate zones, rich vegetation species, and intricate geographic distribution, China ranks among the world’s most biodiverse countries, boasting an extensive wealth of botanical resources. The topography of China exhibits a west-to-east gradient, with elevated terrain in the western regions and lower elevations in the eastern areas [39]. Approximately 67% of the total land area is composed of mountains, plateaus, and hills, while basins and plains comprise the remaining 33% of the land. This topographical diversity contributes to the unique ecological landscapes found throughout the country. On a global scale, China stands as one of the major potential sources of invasive species from the rest of the world, given the existing patterns of invasive species and trade within its borders [40].

### 2.2. Data Resources

Five types of data were collected as shown in Table 1. The FWW occurrence data were extracted from the National Bureau of Forestry and Grassland [41], the China National Knowledge Infrastructure [42], and the Global Biodiversity Information Facility (GBIF) [43]. The grid data of fractional vegetation cover (FVC) and nighttime lights and China’s mountainous zoning vector data were obtained from the National Tibetan Plateau Data Center [44,45,46,47]. The digital elevation model (DEM) data were obtained from the NOAA National Centers for Environmental Information [48]. The land cover data were obtained from the NASA EOSDIS Land Processes Distributed Active Archive Center [49].

#### 2.2.1. Preprocessing of Fall Webworm Occurrence Data

**Figure 1:**
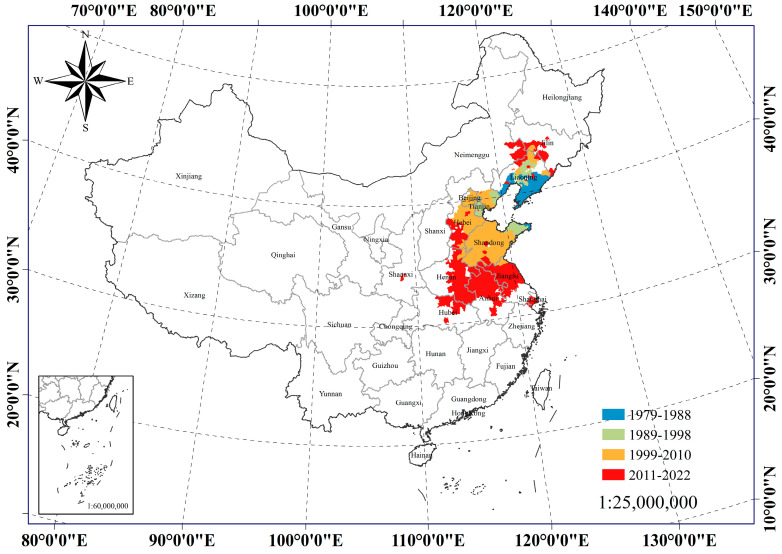
Records of fall webworm epidemic areas in China over time. The blue areas in the figure represent the locations affected by the fall webworm epidemic in China from 1979 to 1988. The green area shows the new epidemic area records of fall webworms in China from 1989 to 1998 compared with the previous period. The orange areas indicate the occurrence of new outbreaks of fall webworms in China from 1999 to 2010 compared to previous outbreaks. The red areas were recorded in China from 2011 to 2022 in comparison to the previous new epidemic areas.

A dataset consisting of 625 occurrences of the FWW was collected in China and subsequently stored in a “.csv” file for analysis. Figure 1 provides a visual representation of the spatial distribution delineating areas affected by the fall webworm. The FWW occurrence data spanned the years from 1979 to 2022 and were divided into four temporal periods (1979–1988, 1989–1998, 1999–2010, 2011–2022) in China. All occurrence points were meticulously verified to ensure that only one point of occurrence per district and county level was retained within the dataset in China. Google Earth was used as the primary tool to obtain the latitude and longitude coordinates of each occurrence point in China [50]. A standardized coordinate system, known as “Krasovsky_1940_Albers”, was utilized consistently throughout this process. Each occurrence point was checked to ensure it had coordinates, and each coordinate was verified to correspond to only one point. Finally, coordinates were obtained for 625 occurrences.

#### 2.2.2. Preprocessing of Topography, Vegetation, and Human Activities Data in China

The preprocessing of grid data, including nighttime lights, DEM, fractional vegetation cover (FVC), and vector data delineating China’s mountainous zones, followed a systematic set of procedures. Initially, each dataset was cropped to align with the geographical extent of China, and a standardized coordinate system, specifically “Krasovsky_1940_Albers”, was uniformly applied. Subsequently, R was used to manipulate the nighttime light data, involving the calculation of average nighttime light values for the years 1984 to 2020. ArcGIS 10.8 was then utilized to conduct statistical analyses within districts and county areas, yielding the average nighttime light data for China’s districts and counties over the specified time period. The FVC dataset of China was derived from 12 composite images, each resulting from the averaging of data spanning eight months (April to November) for each year from 2011 to 2022. Following this, the average values across the 12 images were computed to obtain the FVC averages for districts and counties in China during the years 2011 to 2022. Furthermore, ArcGIS was used for DEM data preprocessing, which involved reclassifying the data into four altitude categories: <0 m, 0–500 m, 500–1000 m, and >1000 m.

### 2.3. Methods

In this study, a variety of methodologies, including the standard deviation ellipse, spatial autocorrelation, and software applications such as ArcGIS 10.8 and R 4.3.1, were utilized for the comprehensive analysis of the spatiotemporal variation of the fall webworm and the factors influencing its dynamics. The invasion dynamics of the black-headed fall webworm (FWW) in China were elucidated by comprehensively considering the transmission path of the black-headed FWW, the spatiotemporal fluctuations observed in the standard deviation ellipse, and the associated center of mass in China. Moreover, the assessment of the various contributing factors to the spatiotemporal changes in fall webworms involved a detailed examination of the digital elevation model (DEM), fractional vegetation cover (FVC), and nighttime light data in China. Consequently, the roles played by topography, vegetation, and anthropogenic factors in shaping the spatiotemporal variations of the black-headed FWW were explained, drawing upon insights from relevant existing studies.

#### 2.3.1. Standard Deviation Ellipse

The standard deviation ellipse (SDE) serves as an effective method to accurately reveal the spatial distribution attributes of geographical elements within spatial statistics. The eccentricity of the spatial distribution analysis (SDE) provides insights into the research object’s distribution pattern [36,37]. Therefore, this paper adopts the standard deviation ellipse method as one of the approaches to elucidate the dynamic pattern of FWW invasion in China. The specific steps are as follows: First, the distribution points of the FWW in different time periods in China are used to construct the SDE of the FWW in different time periods in China by utilizing the SDE tool in ArcGIS software 10.8. Secondly, the spatial and temporal variations of the SDE for the fall webworm (FWW) nationwide were graphically plotted, along with the spatial and temporal variations of the distribution centroid of the FWW during different periods. Thirdly, the spatiotemporal variation of the FWW for China was delineated through the dynamic invasion pattern, incorporating the SDE and centroid data.

#### 2.3.2. Spatial Autocorrelation

Spatial autocorrelation is used to assess the spatial differentiation of research objects by identifying global and local spatial correlation and clustering features within data using the Moran Index [34,35]. It can effectively illustrate the aggregation pattern of nighttime light and vegetation cover across the country. Therefore, using the statistical data of Anselin’s Local Moran’s I implemented in ArcGIS, this paper employs spatial autocorrelation methods to elucidate the impact of human and plant factors on the spread of the FWW. The procedural steps were as follows: Firstly, Anselin Local Moran’s I analysis was performed on the pre-treated nighttime light and FVC data to obtain their county-level aggregation. Then, the resulting Anselin Local Moran’s I analysis results were overlaid with the FWW occurrence points, respectively. This comparison facilitated the interpretation of the influence of both human-related and vegetation-related factors on the diffusion of the FWW.

## 3. Results

### 3.1. Dynamic Invasion Pattern of Fall Webworms in China

#### 3.1.1. Transmission Trajectory of Fall Webworm in China

**Figure 2:**
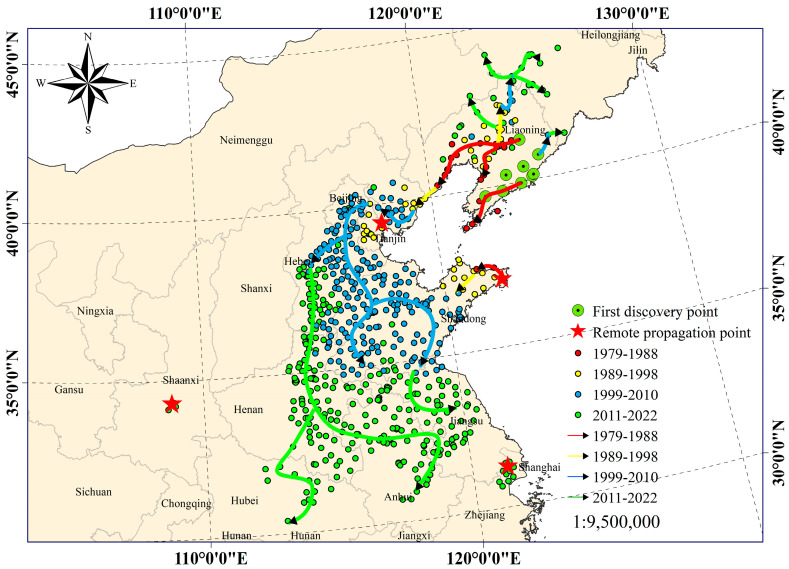
The transmission path of the fall webworm in China over the past 40 years. In the figure, the point of the green circle with the black center represents the areas where the fall webworm epidemic was first discovered in China. The red five-pointed stars filling the dots represent several typical long-distance transmissions that occurred in the history of fall webworm transmission in China. The red dots represent the locations of the fall webworm epidemic in China from 1979 to 1988. The yellow dots represent the new epidemic area records of fall webworms in China from 1989 to 1998 compared to the previous period. The blue dots represent the occurrence of new fall webworm outbreaks in China from 1999 to 2010 compared to previous outbreaks. The green dots were recorded in China from 2011 to 2022 in comparison to the previous new epidemic areas. The red line with arrows shows the transmission path of fall webworms during the period 1979–1988. The yellow line with arrows shows the transmission path of the fall webworm during the period 1989–1998. The blue line with arrows shows the transmission path of fall webworms during the period 1999–2010. The green line with arrows shows the transmission path of the fall webworm during the period 2011–2022.

As depicted in Figure 2, the 40-year dispersal trajectory of the FWW was graphically represented across four distinct periods. The first stage of FWW occurrence spanned from 1979 to 1988 and was confined within the latitudinal range of 37° N to 42° N. During this period, the rate of FWW propagation was characterized by a gradual pace, with a singular point of origin and two distinct propagation directions. The primary propagation direction originated from the initial occurrence zone in Liaoning Province, extending along the coastline and inland within Liaoning Province. The secondary propagation direction involved the transportation of the FWW to Weihai City, Shandong Province, in 1982, facilitated by local fishing boats crossing the Yellow Sea. Subsequently, the infestation progressed northwestward along the coastline to Yantai. 

The second stage of FWW occurrence spanned the period from 1989 to 1998, covering a geographic distribution ranging from 36° N to 43° N. During this period, the rate of FWW propagation exhibited a slight acceleration compared to the preceding period, involving two sources and three distinct propagation directions. The initial propagation direction originated from the FWW occurrence zone in Liaoning Province during the previous stage, spreading northward along the Northeast Plain and covering extensive areas within Liaoning Province. The second propagation direction began from the FWW occurrence area in Liaoning Province during the previous stage, spreading southwestward along the coastline to Qinhuangdao City, Hebei Province. In 1995, the pest was introduced into Tianjin due to long-distance transportation. The third propagation direction originated from Weihai City and Yantai City during the previous stage, extending inland to the west, almost reaching both cities. 

The third stage of FWW occurrence spanned the period from 1999 to 2010 and spread across the range of from 34° N to 44° N. During this period, the rate of FWW propagation increased rapidly compared to the previous period, involving three sources and four propagation directions. The first propagation direction originated from the FWW occurrence in all regions within Liaoning Province in the previous stage, extending along the Northeast Plain to Jilin Province. The second propagation direction began from Qinhuangdao in Hebei Province and extended southwestward along the coastline to Tangshan City. The third wave of transmission started from Tianjin and spread to Beijing, Hebei, Shandong, and other provinces. The fourth propagation direction started from Yantai City in Shandong Province and extended westward to all parts of Shandong Province, spreading along the coastline southward to Lianyungang City in Jiangsu Province. 

The fourth stage of FWW occurrence spanned the period from 2011 to 2022, distributed within the range of 30–45° N. During this period, the rate of FWW occurrence was the fastest since its invasion of China. There were two sources and three propagation directions. The first propagation direction originated from all areas east of Shanhai Pass in the third stage and extended to more areas in Northeast China. The second propagation direction began from the third stage occurrence zone in Hebei and Shandong provinces, spreading southward to most areas in Jiangsu, Anhui, Henan, and other provinces. It was also introduced by long-distance transport to the coastal city of Shanghai. The third propagation direction originated from the occurrence zone in Hebei and Shandong provinces and spread through long-distance transport to the inland city of Shaanxi in the west. Based on this analysis, we concluded that the dynamic invasion pattern of the FWW in China observed from 1979 to 2022, followed a sequence of “invasion-occurrence-propagation-outbreak” patterns from coastal to inland regions. 

#### 3.1.2. Spatiotemporal Variation of Standard Deviation Ellipse in China

**Figure 3:**
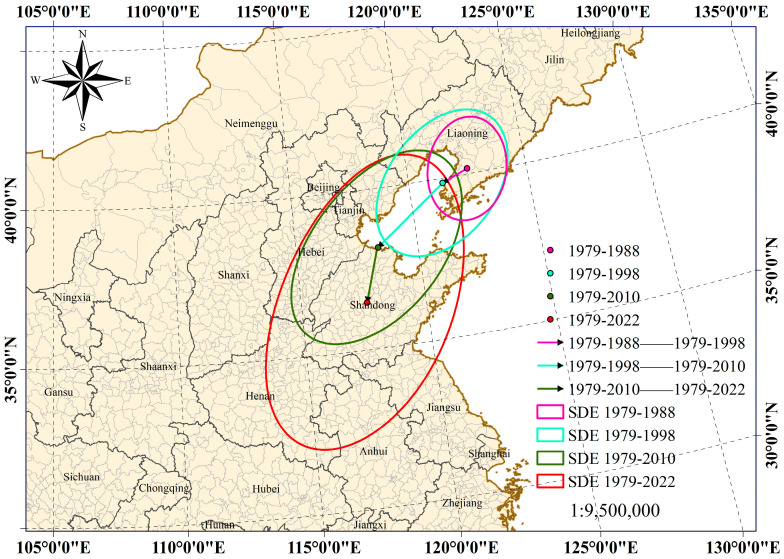
Spatiotemporal variation of the SDE and centroid of the national fall webworm over the past 44 years. In the figure, the magenta dots indicate the centers of mass of all fall webworm epidemic areas during the period 1979–1988. The cyan dots indicate the centroid of all epidemic areas of the FWW from 1979 to 1998. The green dots indicate the centroids of all epidemic areas of the FWW from 1979 to 2010. The red dots indicate the centers of mass of all epidemic areas of the FWW from 1979 to 2022. The magenta line with an arrow represents the direction of movement from the centroid of all epidemic areas of the FWW during the period 1979–1988 to the centroid of all epidemic areas of the FWW during the period 1979–1998. The cyan lines with arrows represent the direction of movement from the centroid of all epidemic areas of the FWW from 1979 to 1998 to the centroid of all epidemic areas of the FWW from 1979 to 2010. The green lines with arrows represent the direction of movement from the centroid of all epidemic areas of the FWW during the period 1979–2010 to the centroid of all epidemic areas of the FWW during the period 1979–2022. The magenta circles represent the SDE of the FWW during the period 1979–1988. The cyan circles represent the SDE of the FWW during the period 1979–1998. The green circles represent the SDE of the FWW for the period 1979–2010. The red circles represent the SDE of the FWW for the period 1979–2022.

We analyzed the spatial distribution of the SDE and its centroid over different time periods, taking into account the occurrences of the FWW in epidemic areas nationwide. This comprehensive investigation enabled us to derive insights into the alterations in SDE and centroid positioning of the FWW throughout the 44-year duration (Table 2, Figure 3).

As evident in Figure 3, it can be deduced that since the earliest period under investigation, the spatial distribution of the FWW in the country has followed a “southwest to northeast” directional pattern, progressively inclining towards the south over time. Additionally, the westward spread has gradually slowed down. The eccentricity of the FWW across the entire country exhibited a diminishing trend across different time periods, indicative of a gradual flattening in the spatial distribution shape of the FWW. This transformation was accompanied by an expansion of the FWW in the southwest direction. Over the past four decades, the FWW has demonstrated a significant shift in its center of gravity, starting at 122.5379° E, 40.1688° N during the first period and subsequently moving southwestward to 121.4202° E, 39.8537° N. This southwesterly displacement continued, reaching 118.3715° E, 38.1492° N and ultimately culminating in a further shift to 117.6351° E, 36.4863° N. The analysis of the centroids’ distribution across all four periods highlights the predominant southward trajectory of FWW dispersal. Specifically, from the initial period to the present, the centroid has shifted southward by 365.1 km and westward by 460.6 km. The westward travel distances of E_1_–E_2_ and E_2_–E_3_ were greater than the southbound propagation distances, which were more than 70.2 km and 125.0 km, respectively. The distance to the west of E_3_–E_4_ was much smaller than the distance to the south, which was 99.7 km (Table 2, Figure 3).

### 3.2. Analysis of the Influencing Factors

#### 3.2.1. Human-Related Factor

**Figure 4:**
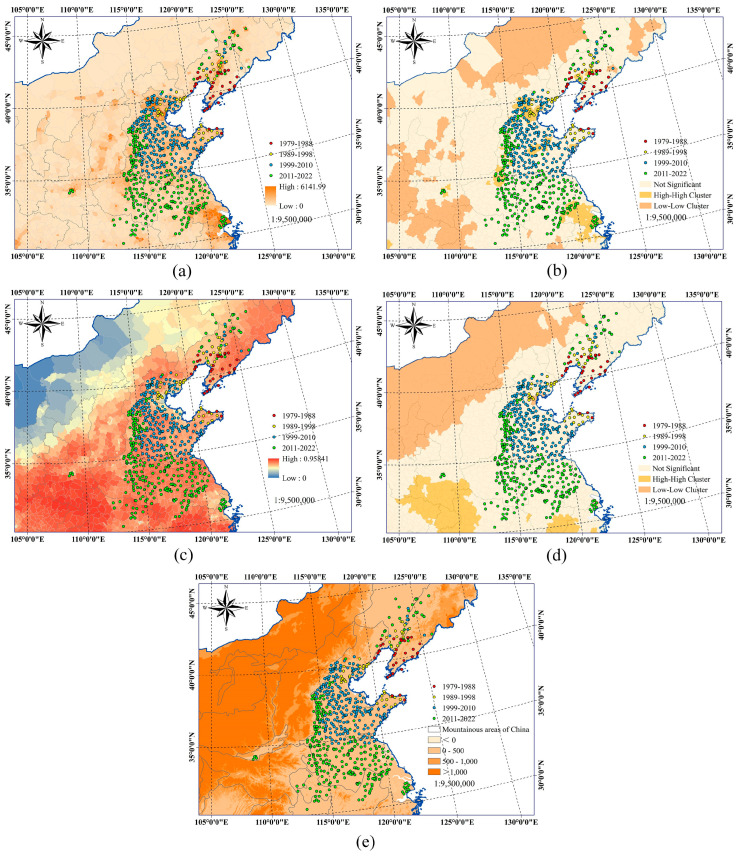
Spatial relationships between humans, vegetation, topography, and the fall webworm in China. In (**a**–**e**), the red dots represent the records of the fall webworm epidemic area in China from 1979 to 1988. The yellow dots indicate the new epidemic area records of fall webworms in China from 1989 to 1998 compared to the previous period. The blue dots show the records of new outbreaks of fall webworms in China from 1999 to 2010 relative to previous outbreaks, and the green dots represent the records from 2011 to 2022 in China relative to the previous new epidemic areas. The specific introduction is as follows: (**a**) Spatial relationship between nighttime light data and the fall webworm by district and county in China. In (**a**), numbers 0 to 6141.99 represent the size of the background DN value, which indicates the intensity of nighttime light in various districts and counties. The darker the color, the stronger the nighttime light, and the lighter the color, the weaker the nighttime light. (**b**) Spatial autocorrelation data of nighttime light and the spatial relationship of fall webworms in China. In (**b**), the “High-High Cluster” represents areas with highly concentrated nighttime light, indicating that the nighttime light in these areas and surrounding counties was all high. The “Low-Low Cluster” refers to an area with low nighttime light, meaning that both the area and its surrounding counties experience low nighttime light levels. The term “Not Significant” indicates that there was no significant relationship between the nighttime light levels of these counties and the surrounding areas, and no distinct group of areas with higher or lower nighttime light values was formed. (**c**) Spatial relationship between fractional vegetation cover (FVC) data and fall webworms by district and county in China. In (**c**), numbers 0 to 0.9584 indicate the extent of the background vegetation coverage index, representing the vegetation coverage size in various districts and counties. The darker the color, the richer the vegetation coverage, and the lighter the color, the sparser the vegetation coverage. (**d**) Spatial autocorrelation data of fractional vegetation cover (FVC) and the spatial relationship of fall webworms in China. In (**d**), the “High-High Cluster” represents areas with highly concentrated FVC, indicating that the FVC of these areas and surrounding counties were all high. The “Low-low Cluster” indicates areas with low FVC, meaning that the FVC in these areas and surrounding counties was all low. The term “Not Significant” indicates that there was no significant relationship between the FVC of these counties and the surrounding counties, and no distinct group of areas with higher or lower FVC values was formed. (**e**) The spatial relationship between the digital elevation model (DEM) and mountainous regions with fall webworms in China. In (**e**), the background was divided into four ranges according to the depth of color, which were successively <0 m, 0–500 m, 500–1000 m, and >1000 m. The term “Mountainous areas of China” refers to the geographical range of all mountainous regions in China.

As shown in Figure 4a,b, during the period from 1979 to 1988, the majority of FWW occurrence zones were located in areas with low nighttime light values and were not significant cluster areas. During the period from 1989 to 1998, the major regions where FWW spread featured low nighttime light values and were non-concentrated, while the long-distance transmission region (Tianjin) offered high nighttime light values and was highly concentrated. In the period spanning 1999 to 2010, the nighttime light value of the new FWW occurrence zones was generally higher than that of the previous two periods. Finally, from 2011 to 2022, the nighttime light values in the new FWW occurrence zones were similar to those in the previous period. The two long-distance transmission points in the Shanghai and Shaanxi regions exhibited high nighttime light values and were highly concentrated. This suggests that the black-headed FWW is more likely to occur in areas with high nighttime light values.

#### 3.2.2. Topographical and Vegetation Factors

Figure 4e indicates that in China, the elevation of the four-period FWW occurrence zone was below 500 m, with a small fraction occurring between 500 and 1000 m. Since its introduction from northern China into Hebei and Tianjin, the black-headed FWW has not spread westward to areas with elevations exceeding 1000 m, where climatic conditions are suitable for its survival. Instead, it has spread to areas southwest and southward with elevations below 500 m. The FWW has invaded Hebei and Tianjin for over 20 years. The pest has not shown severe damage in areas above 500 m in altitude, primarily affecting regions below this threshold. Furthermore, Figure 4c,d, and Figure S1 show that in China, the four-period FWW occurrence zones exhibit little variation in the FVC values and do not indicate significant cluster areas. A limited number of FWW occurrence zones were revealing low-low cluster areas with low vegetation coverage. The FWW had not caused severe destruction in high-high cluster areas with dense vegetation coverage and forest areas. Even if some occurred in areas with high vegetation cover, these areas are less than 500 m above sea level and are non-forested.

## 4. Discussion

### 4.1. Dynamics of Fall Webworm Invasion in China

Our investigation reveals the dissemination pattern of the black-headed FWW between 1979 and 2022. It is characterized by a sequence of “invasion-occurrence-transmission-outbreak”, progressively spreading from coastal to inland regions. In the first stage of invasion, the FWW showed slow propagation characteristics and long-distance transmission events occurred from Dalian to Weihai by fishing boats [16]. This may be attributed to the initial invasion phase when the pest is acclimating to the local environment and identifying suitable hosts. Throughout this period, the temperature in Liaoning Province remained notably low, which allowed this pest to survive only in adjacent regions, while preventing its natural spread to neighboring areas [51,52].

In the second phase of the invasion, the FWW exhibited a swifter spread compared to the initial phase, with localized expansion observed within Liaoning Province and further dissemination southwestward to Qinhuangdao, Shandong Province. Additionally, a long-distance transmission event was documented from Liaoning Province to Tianjin. This change can be attributed to the pest’s improved adaptation to the local environment and the formation of a stable population, which facilitates rapid spread. The third phase of transmission exhibited an exceptionally rapid pace, covering almost all regions of Hebei, Shandong, and Beijing. These areas are primarily located within the North China Plain, characterized by relatively flat terrain, low elevation, and the absence of significant mountainous barriers. Such geographical features, coupled with more favorable climatic conditions compared to the Northeast, contributed to a marked swifter spread across the North China Plain during this specific stage of the invasion, in contrast to the Northeastern region.

In the fourth phase, the pest rapidly spread to the central and eastern regions, with two long-distance transmission events occurring. The accelerated spread of the FWW during this fourth invasion stage can be attributed to several factors. Firstly, economic growth can foster increased transportation and trade activities, inadvertently aiding the FWW in its expansion into new areas. Secondly, the favorable climatic conditions in the central and eastern regions of China may have provided conducive environments for the pest’s survival and dissemination [53]. Research by Lu et al. [54] supports this notion, indicating a gradual increase in the generational quantity of the FWW in China from north to south.

Moreover, drawing from the dynamic change in the standard deviation ellipse of the FWW, it becomes evident that over the past 44 years, the spatial distribution of the FWW in China has exhibited a southwest–northeast directional pattern that has gradually tilted towards the southwest. Notably, the center of gravity of the FWW has shifted significantly. Specifically, the westward propagation distance between E_1_–E_2_ and E_2_–E_3_ was greater than the southward propagation distance, which was over 70.2547 km and 125.0088 km, respectively. The distance to the west of E_3_–E_4_ was much smaller than the distance to the south, at 99.7238 km (Table 2, Figure 3). It means that the shift of the centroid of the E_1_–E_2_ and E_2_–E_3_ distributions to the west was significantly greater than the shift to the south. However, the movement of the distribution centroid of E_3_-E_4_ significantly slowed down to the west while also showing a notable shift to the south. This observation highlights a clear trend of the FWW spreading further south from current epidemic areas.

In summary, we found that the transmission of the FWW in China is characterized by an “invasion-occurrence-transmission-outbreak” sequence. It gradually spreads from coastal areas to inland areas, with a noticeable trend in areas south of 30° N in southern China and north of 45° N in northern China, particularly in the southern region. This observed pattern resonates with earlier predictions about FWW dispersion within the Chinese territory [55]. The trajectory of transmission aligns with the findings reported by Dai et al. [22], underscoring a consistent and congruent trend in FWW dispersion within the region.

### 4.2. Effects of Human-Related Factors on the Dynamic Invasion of the Black-Headed Fall Webworm

Human activities can significantly influence the population distribution patterns of non-native and invasive insects that are inadvertently introduced into new environments. Factors such as gross domestic product (GDP), introduction pressure, and the importation of goods can contribute to the proliferation of invasive alien species within a given region or country [56]. The spread of the FWW in China has gradually extended to areas characterized by high nighttime light values over the past four decades, especially in regions with long-distance transmission along the coast (such as Tianjin and Shanghai). In these areas, FWW occurrences were documented one or more years before their official detection, and most of them were initially discovered in areas with local ports. The reason for this is that the insect exhibits strong phototaxis, which attracts it to cargo ships in transit and facilitates its spread to new areas. The expansion of nighttime lights is a crucial indicator of urban economic prosperity, reflecting both economic and social aspects. Higher nighttime light levels signify a denser population, increased traffic intensity, and more concentrated land use. In such areas, invasive species are also more easily and frequently detected. In other words, population density is a primary factor that positively influences night-time light intensity, traffic intensity, the thoroughness of the area investigation, as well as the spread (local introductions) of invasive species [57,58]. Spatial analysis of night light data in China and the occurrence of the FWW showed that the FWW was more prevalent in areas with higher night light values in China (Figure 4). It is worth noting that since the discovery of the novel coronavirus in China in December 2019, human activities in the country have experienced a decline due to lockdown and quarantine policies, resulting in reduced demand and stalled capital flows [59]. This indicates that after the implementation of quarantine policies in China, human-related factors had a minimal impact on the FWW. Nevertheless, the FWW continues to spread naturally under the influence of other factors. The maximum cumulative flight distance of each female was 8.21 km [60]. Spontaneous (local) population densification and dispersal may be hindered by mountains or unsuitable vegetation cover. Despite mountains and unsuitable vegetation cover, they cannot block further long-distance anthropogenic transport. However, a meticulous examination of FWW occurrence sites reveals a deviation from the previous trend of an average annual increase of 30 new outbreak areas before 2019, which also included cases of long-distance transmission. Specifically, there were seven new outbreak areas in 2020, three in 2021, and two in 2022. Overall, these data indicate the crucial role of human-related factors in the dissemination of this pest.

### 4.3. Effects of Topographical and Vegetation-Related Factors on the Dynamic Invasion of the Black-Headed Fall Webworm

Geographical factors exert a significant influence on insect transmission, and the contiguous landmass serves as a barrier to species distribution, particularly with the presence of high undulating mountains providing a strong elevation zonation for plants and animals [61]. The survival of herbivorous insects is intricately linked to the presence of host plants, where the quality of the host plant becomes a key determinant of their fecundity. Carbon, nitrogen, and defensive metabolites in the host plant directly impact the fertility of these herbivorous insects [62]. FVC, defined as the proportion of green vegetation visible from the lowest point, is a crucial variable for characterizing the status of surface vegetation [63]. Our study reveals that in China, the spread of the FWW was hindered by mountainous terrain. Almost all FWW incidents occurred at altitudes below 500 m in non-forested areas. In regions with high vegetation coverage, the presence of the pest was notably absent. Furthermore, according to previous research, vegetation serves as an indispensable food source for herbivorous insects, and the severity of infestation increases with greater host abundance [64]. Although the black-headed FWW has caused detrimental impacts in China, these regions have low vegetation coverage. Even in areas with higher vegetation coverage, the elevation is less than 500 m, and they are situated in non-forest regions. This is similar to the findings of previous studies, indicating that in Japan and South Korea, the black-headed FWW primarily occurs in urban areas and has not been detected in forests [65]. In summary, the black-headed FWW is more likely to occur in non-forested areas with low vegetation cover at altitudes below 500 m in China.

### 4.4. Recommendations for Management and Prevention of FWW

In terms of human activity, the black-headed FWW is more likely to spread in areas with high nighttime light values and areas with abundant ports. In terms of topography and vegetation, the black-headed FWW is more likely to occur in non-forested areas with low vegetation cover at altitudes below 500 m. There was an evident trend for the pest to spread to the southern regions of China. If the insect extends its range to more southern regions such as Guangdong and Hainan, where the climate is conducive to multiple generations each year, it could pose a significant threat to plant resources in southern China. Initially, it is necessary to enhance monitoring of cities surrounding the affected areas with high nighttime light values, relatively flat terrain, elevations below 500 m, and limited vegetation coverage. Second, the coastal cities along the Yangtze and Yellow Rivers in non-epidemic areas, particularly cities with significant ports (such as Taizhou and Wenzhou in Fujian Province), should enhance the quarantine measures for imported goods. In addition, monitoring of pests in inland cities in non-epidemic areas must be strengthened, especially in non-forested areas (such as the Sichuan Basin) where the climate is suitable for pest growth, the elevation is less than 500 m, and vegetation cover is limited.

## 5. Conclusions

In this study, we revealed that the transmission of the FWW in China is characterized by an “invasion-occurrence-transmission-outbreak” sequence, gradually spreading from coastal areas to inland areas. There was an obvious spreading trend in the southern region. The FWW was more likely to spread in areas with high nighttime light values, abundant ports, and non-forested areas with low vegetation cover at altitudes below 500 m.

## Figures and Tables

**Table 1:** The definition and source of each data.

Data Type	Data	Definition	Source
Occurrence data	Occurrence Point Data	Occurrence point data of the FWW between 1979–2022 in different regions of China	The National Bureau of Forestry and Grassland, the China National Knowledge Infrastructure, and the Global Biodiversity Information Facility (GBIF)
Human data	Nighttime Lights Data	A Prolonged Artificial Nighttime-light Dataset of China (1984–2020)	The National Tibetan Plateau Data Center
Vegetation data	Fractional Vegetation Cover	China regional 250 m fractional vegetation cover dataset (2000–2022)	
Topography data	China’s Mountainous Zoning	Dataset of “Digital Mountain Map of China” (2015)	
	Digital Elevation Model (DEM)	ETOPO 2022 15 Arc-Second Global Relief Model	NOAA National Centers for Environmental Information
Land use data	Land Cover	MODIS/Terra+Aqua Land Cover Type Yearly L3 Global 500 m SIN Grid	NASA EOSDIS Land Processes Distributed Active Archive Center

**Table 2:** Centroid travel distance of fall webworms in the past 44 years. E_1_ was the centroid of all epidemic areas of the FWW in China during 1979–1988. E_2_ was the center of all epidemic areas of the FWW in China during 1979–1998. E_3_ was the centroid of all epidemic areas of the FWW in China during 1979–2010. E_4_ was the centroid of all epidemic areas of the FWW in China from 1979 to 2022. The time intervals of the four centroid transition events E_1_–E_2_, E_2_–E_3_, E_3_–E_4_, and E_1_–E_4_ are 10, 12, 12, and 34 years, respectively.

Centroid Transfer Event	Total Linear Distance (km)	Annual Mean Distance (km)	Westward Drift (km)	Annual Mean Westward Drift (km)	Southward Drift (km)	Annual Mean Southward Drift (km)
E_1_–E_2_	101.6	10.2	97.8	9.8	27.6	2.8
E_2_–E_3_	324.8	27.1	283.5	23.6	158.5	13.2
E_3_–E_4_	195.8	16.3	79.3	6.6	179.0	14.9
E_1_–E_4_	592.2	17.4	460.6	13.5	365.1	10.7

## Data Availability

The datasets generated in this study are available upon request from the corresponding authors.

## Supplementary Materials

The following supporting information can be downloaded at: https://www.mdpi.com/article/10.3390/insects15050349/s1, Figure S1: Spatial distribution map of land cover types at FWW sites in China.
