# Supplementary material for: Exploring the Dynamic Invasion Pattern of the Black-Headed Fall Webworm in China: Susceptibility to Topography, Vegetation, and Human Activities
Source: Insects. 2024 May 13;15(5):349. doi: 10.3390/insects15050349 (PMC11121765; doi:10.3390/insects15050349)

Figure S1. Spatial distribution map of land cover types at FWW sites in China. The "Non-forested area" were 6-14 and 16 of the 17 types defined by International Geosphere-Biosphere Programme (IGBP) in MCD12Q1_V61 products. The "Forest area" were 1-5 of the 17 types defined by IGBP in MCD12Q1_V61 products. The "Water area" were 15 and 17 of the 17 types defined by IGBP in MCD12Q1_V61 products.

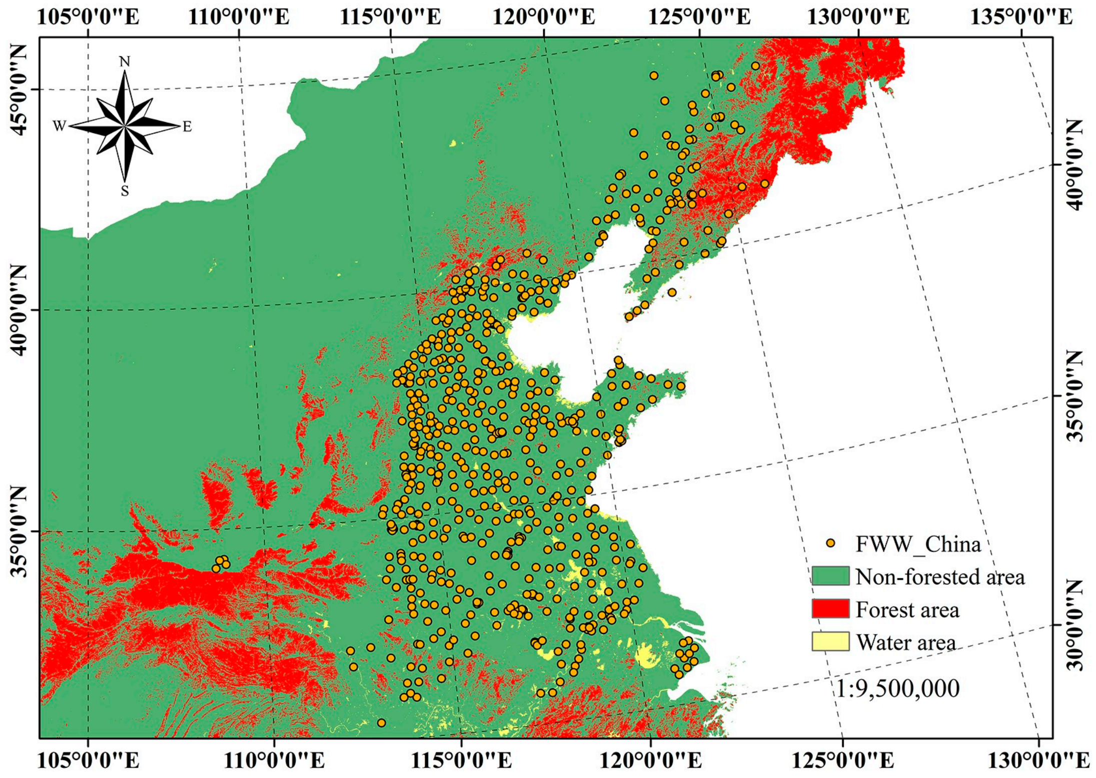

Supplement: Supplementary file 1 [file insects-15-00349-s001.zip › insects-2952403-supplementary.pdf]
